# One-Shot, One Opportunity: Retrospective Observational Study on Long-Acting Antibiotics for SSTIs in the Emergency Room—A Real-Life Experience

**DOI:** 10.3390/pathogens14080781

**Published:** 2025-08-06

**Authors:** Giacomo Ciusa, Giuseppe Pipitone, Alessandro Mancuso, Stefano Agrenzano, Claudia Imburgia, Agostino Massimo Geraci, Alberto D’Alcamo, Luisa Moscarelli, Antonio Cascio, Chiara Iaria

**Affiliations:** 1 Infectious Diseases Unit, ARNAS Civico-Di Cristina Hospital, 90100 Palermo, Italy; 2 Infectious Diseases Unit, Umberto I Public Hospital, 96100 Siracusa, Italy; 3 Emergency Room, ARNAS Civico-Di Cristina Hospital, 90100 Palermo, Italy; 4 Pharmacy Unit, ARNAS Civico-Di Cristina Hospital, 90100 Palermo, Italy; 5 Infectious Diseases Unit, P. Giaccone University Hospital, 90100 Palermo, Italy

**Keywords:** long-acting antibiotics, emergency room, dalbavancin, oritavancin, skin and soft tissue infections

## Abstract

Background: Skin and soft tissue infections (SSTIs) are a major cause of emergency room (ER) visits and hospitalizations. Long-acting lipoglycopeptides (LALs), such as dalbavancin and oritavancin, offer potential for early discharge and outpatient management, especially in patients at risk for methicillin-resistant *Staphylococcus aureus* (MRSA) or with comorbidities. Methods: We conducted a retrospective observational cohort study from March to December 2024 in an Italian tertiary-care hospital. Adult patients treated in the ER with a single dose of dalbavancin (1500 mg) or oritavancin (1200 mg) for SSTIs were included. Demographic, clinical, and laboratory data were collected. Follow-up evaluations were performed at 14 and 30 days post-treatment to assess outcomes. Results: Nineteen patients were enrolled (median age 59 years; 53% female). Most had lower limb involvement and elevated inflammatory markers. Three patients (16%) were septic. Fourteen patients (74%) were discharged without hospital admission; hospitalization in the remaining cases was due to comorbidities rather than SSTI severity. No adverse drug reactions were observed. At 14 days, 84% of patients had clinical resolution; only 10% had recurrence by day 30, with no mortality nor readmission reported. Conclusions: LALs appear effective and well-tolerated in the ER setting, supporting early discharge and reducing healthcare burden. Broader use may require structured care pathways and multidisciplinary coordination.

## 1. Introduction

Skin and soft tissue infections (SSTIs) represent a significant burden on access to emergency rooms (ERs) [1] and hospital admissions [2].

The place in therapy of long-acting lipoglycopeptides (LALs), namely dalbavancin and oritavancin, to treat serious SSTIs in the ER is challenging but represents an opportunity to reduce hospital admissions and their relative costs and mortality [3,4]. Rates of community-acquired methicillin-resistant *Staphylococcus aureus* (CA-MRSA) SSTIs vary worldwide [5], ranging from 1% to 50%. Furthermore, patients with MRSA SSTIs (especially those with comorbidities) may experience bacteremia with longer in-hospital stays and a higher risk of mortality [5]. LALs are approved for acute bacterial skin and skin-structure infection (ABSSSI), but their place in therapy among ER patients is not clear. On the other side, LALs could be used in ERs to avoid hospital admission, resulting in high cost-efficacy [6]. Nevertheless, international guidelines on SSTIs from the Infectious Diseases Society of America (IDSA) [7] are dated and do not include LALs; instead, the World Society of Emergency Surgery (WSES) [8] includes only dalbavacin for the treatment of SSTIs. Therefore, we would describe our nine-month real-life experience with LALs in the emergency department of a tertiary-care hospital to evaluate the impact on discharge rate, mortality, recurrence and readmission among LAL-treated patients.

## 2. Materials and Methods

This study was conducted in accordance with the Helsinki Declaration, and patients’ data were collected anonymously. We conducted a retrospective observational cohort study. The observational period ranged from March to December 2024 in an Italian tertiary-care hospital. Data were collected retrospectively, analyzing electronic medical records (EMR) of patients who were prescribed dalbavancin 1500 mg or oritavancin 1200 mg one-shot in the ER (all the treatments were given during the first 48 h−72 h in the ER) defined as per IDSA guidelines, namely cellulitis and erysipelas [7]. Patients were included if they were (1) ≥18 years old, (2) admitted to the Emergency Unit due to an SSTI, and (3) evaluated by an infectious disease (ID) specialist who prescribed and administered LALs (dalbavancin 1500 mg or oritavancin 1200 mg one-shot).

Patients were excluded if they were (1) <18 years old, (2) pregnant, (3) in septic shock, or (4) with a life expectancy of ≤6 months. The STROBE checklist is provided in Supplementary S1.

Clinical data were collected at in-hospital admission (comorbidities and Charlson Comorbidity Index—CCI, sex, age, antibiotics prior to and in association with the type of LAL (Supplementary S2), body temperature, heart rate—HR, Glasgow Coma Scale—GCS, arterial pressure, SOFA score, peripheral saturation—SpO2, and oxygen inspired fraction—FiO2) together with a blood test examination (white blood cells—WBC, C-reactive protein—CRP, procalcitonin—PCT, creatinine, and bilirubin) upon arrival to the ER.

In the ER, patients were re-evaluated by ID and ER specialists during the first 24 h−48 h after LAL administration to reach a decision about whether or not to discharge the patients.

Follow-up visits at 14 days ± 5 days and 30 days ± 7 days after LAL administration were also included to confirm 14-day healing (based on clinical judgment and blood test examination) and 30-day recurrence, readmission and mortality. Given the retrospective nature of the study, a follow-up blood test examination was not included due to the different timing of blood sampling and the varying types of tests requested. Data were analyzed using SPSS ver. 21©.

Dichotomous variables are presented as a numerator and denominator, along with the percentage. Continuous variables are shown as median and interquartile (IQR) range 25−75% with a 95% Confidence Interval (CI).

Dichotomous variables were analyzed using Fisher’s exact test (two-tailed), continuous variables were analyzed using the Mann–Whitney U test, and the *p*-value was considered significant if it was ≤0.05.

## 3. Results

We included 19 patients; the median age was 59 (IQR, 45–74); most of them were females, 10/19 (52.6%). All 19 patients were treated with LALs during ER admission, had a GCS of 15, and no patients met the exclusion criteria. In most cases, the lower extremities were involved, 15/19 (78.9%). The majority of patients, 11/19 (57.9%), had a short antibiotic course before ER admission (less than 4 days), detailed in Supplementary S2.

None of the patients in our cohort were affected by heart failure, CKD (chronic kidney disease), cerebral vasculopathy, or AIDS, and the GCS was 15 for all patients.

The most common comorbidity was peripheral vasculopathy (58%), followed by chronic pulmonary diseases (26%). Other comorbidities, such as dementia (10.5%) or liver diseases (10.5%), were also found. The rates of type II diabetes (10.5%), neoplasm (10.5%), and chronic heart disease (5%) were similar to the non-SSTI population. The median CCI was 3 (IQR, 1–5), with higher points among hospitalized patients, a CCI of 5 (IQR, 2–5.5), compared to non-hospitalized patients with a CCI of 3 (IQR, 1–5), but not statistically significant (*p* = 0.56). Only one patient had missing data for HR, body temperature, bilirubin, and PCT.

In our cohort, dalbavancin and oritavancin were administered in similar percentages (58% vs. 42%, respectively). No adverse drug reaction (ADR) was found. Overall, 14/19 (73.7%) patients were discharged from ER, and 5/19 (26.3%) were hospitalized due to comorbidities: two patients with a suspicious neoplasm, one patient due to frailty, one patient due to deep vein thrombosis, and one patient due to sepsis (a systemic response with high inflammatory marker levels, hypotension and acute respiratory failure). A comparison of the baseline characteristics between hospitalized and discharged patients was performed; no statistically significant differences were found between the two groups (Table 1).

Note that our population has high inflammatory marker levels, with WBC at 11,900 cells/uL (IQR, 8800–16,400), C-reactive protein at 14.6 mg/dL (IQR, 7.1–19.6), and PCT at 0.3 ng/mL (IQR, 0.07–1.1), with higher levels among the “hospitalized” group.

Among our cohort, 3/19 (15.8%) patients were septic (SOFA = 2); they all recovered, but one of them experienced a 30-day recurrence.

Overall, only 5/19 (26%) of patients needed adjunctive antibiotic therapy after LAL administration, mainly among hospitalized patients, 3/5 (60%), instead of discharged patients, 2/14 (14%).

At the 14-day follow-up examination, only 3/19 (15.8%) patients had local signs of inflammation and pain, but by the 30-day visit, they all had recovered.

Only 2/19 (10.5%) experienced a recurrence; all those patients were considered healed at the 14-day visit. All patients were alive at the 14-day and 30-day visits without hospital readmission.

## 4. Discussion

SSTIs are mostly caused by Gram-positive bacteria [9], and international guidelines recommend treatment with narrow-spectrum antibiotics [7,8]. Considering the high level of MRSA reported in our country [10], our empirical therapy may include anti-MRSA activity, especially in patients with comorbidities.

Given the retrospective nature of our study, selection and performance biases should be taken into account, as should the lack of the blinding of the outcomes. Given the small group of patients in our cohort, statistical calculations could be carefully analyzed.

However, since it is the first real-life study among ER-treated patients with LAL for a SSTI, evaluating both healing, mortality, readmission and recurrence at 14 and 30-day, this study could provide clinicians with some useful information.

Dalbavancin and oritavancin demonstrated clinical non-inferiority in SSTI therapy [11,12]. In our cases, the choices among them were based on the clinician’s decision as well as drug availability. In our cohort, 58% of patients failed with previous short antibiotic treatment, making an ER admission necessary, highlighting the high efficacy of LALs also in patients at risk of multi-drug resistant (MDR) pathogens.

The high rates of peripheral vasculopathy (58%) and chronic pulmonary diseases (26%) may induce thinking about the importance of good tissue oxygenation to avoid SSTIs. Furthermore, patients with comorbidities are at risk of SSTIs, as demonstrated by our median CCI of 3 (1–5). The high inflammatory marker levels in our cohort (note, two patients needed supplementary oxygen therapy in the ER for a sudden onset of respiratory failure) make it an ideal (real-life) population for evaluating the efficacy of both drugs.

Despite this, 74% of patients were discharged after a few days in the ER (median of 3 days), and the 14-day response was highly favorable, with 84% of patients healed. Therefore, LALs are a strong and well-tolerated option (no ADR in our cohort) to treat SSTIs in the ER; their benefit, the rapid discharge of the patient and a good clinical response, could outweigh the price. Also, in the case of patients at risk of MDR (or that previously failed antibiotic treatment), septic patient or with a systemic inflammatory response, the long half-life and the excellent safety profile make LALs ideal agents for managing even severe SSTIs in an outpatient setting, making them a real game-changer in this field.

## 5. Conclusions

The first issue we encountered was the lack of standardized criteria to identify patients who require hospitalization or emergency care treatment for SSTIs. The difficulty of discharging ill patients with SSTIs was eased by their rapid clinical improvement with LALs, the sharing of decision-making and responsibility between ER and ID specialists, and the short follow-up period. Moreover, to implement LALs as a standard of care in our ER, we needed the prompt availability of an ID consultant and 24/7 access to LAL treatment.

LALs may contribute as a stewardship strategy, dramatically reducing the length of hospitalization and healthcare-related infections: the majority of patients were early discharged, and most of them did not need antibiotic co-medication.

Early discharge is a cost-effective strategy [6], but it also represents a governance strategy in an overwhelmed healthcare system where resources are scarce.

To implement LALs in a healthcare pathway, we need to encourage multidisciplinary teamwork and to improve telemedicine follow-up programs.

## Figures and Tables

**Table 1:** Baseline and follow-up patients’ characteristics.

	Overall (n = 19)	Hospitalized (n = 5)	Discharged (n = 14)	*p*-Value
Sex (male)	9/19 (47.4%)	1/5 (20%)	8/14 (57.1%)	0.3
Age	59 (45–74)	71 (40.5–79.5)	58.5 (45–71)	1
Comorbidities				
CHD	1/19 (5.3%)	0/5 (0%)	1/14 (7.1%)	1
Peripheral vasculopathy	11/19 (57.9%)	2/5 (40%)	9/14 (64.3%)	0.6
Dementia	2/19 (10.5%)	1/5 (20%)	1/14 (7.1%)	0.47
Chronic pulmonary disease	5/19 (26.3%)	2/5 (40%)	3/14 (21.3%)	0.57
Type II diabetes	2/19 (10.5%)	1/5 (20%)	1/14 (7.1%)	0.47
Neoplasm	2/19 (10.5%)	1/5 (20%)	1/14 (7.1%)	0.47
Liver disease	2/19 (10.5%)	0/5 (0%)	2/14 (14.2%)	1
CCI	3 (1–5)	5 (2–5.5)	3 (1–5)	0.56
Days in ER	-	-	3 (2–4)	
Days in Hospital	-	22 (5–43.5)	-	
Heart rate ^1^	86 (80–99.3)	88 (78.5–97)	86 (80–100)	0.6
Body temperature ^1^	36.8 (36–38)	37.3 (36–38)	36.6 (36–38.2)	1
WBC (cells/uL)	11,900 (8800–16,400)	16,200 (9500–26,450)	11,250 (8500–15,575)	0.23
PTL (cells/uL)	229,000 (172,000–257,000)	250,000 (212,000–26,450)	226,500 (158,500–255,500)	0.26
Creatinine (mg/dL)	0.84 (0.64–0.97)	0.83 (0.61–0.96)	0.84 (0.68–1)	0.5
Total Bilirubin ^1^ (mg/dL)	0.54 (0.37–0.93)	0.61 (0.49–1.3)	0.52 (0.32–0.8)	0.29
CRP (mg/dL)	14.6 (7.1–19.6)	14.9 (5.7–27)	14.3 (6.9–21.7)	0.89
PCT ^1^ (ng/mL)	0.3 (0.07–1.1)	0.53 (0.2–8.8)	0.25 (0.07–0.93)	0.44
Previous ATB	11/19 (57.9%)	2/5 (40%)	9/14 (64.3%)	0.6
Type of LAL (Dalbavancin)	11/19 (57.9%)	3/5 (60%)	8/14 (57.1%)	1
LAL in combination	5/19 (26.3%)	3/5 (60%)	2/14 (14.2%)	0.08
14-day visit (healing)	16/19 (84.2%)	4/5 (80%)	12/14 (85.7%)	1

^1^ Data missed for one patient. Data are shown as numerators/denominators (percentages) or medians (IRQ 25–75%, 95% CI). CHD: chronic heart disease. CCI: Charlson comorbidities index. ER: emergency room. WBC: white blood cells. PTL: platelets. CRP: C-reactive protein. PCT: procalcitonin. ATB: antibiotic. LAL: long-acting lipoglycopeptide.

## Data Availability

Data is contained within the article or Supplementary Materials.

## Supplementary Materials

The following supporting information can be downloaded at: https://www.mdpi.com/article/10.3390/pathogens14080781/s1. Supplementary S1: STROBE check-list. Supplementary S2: Previous and combination antibiotic treatment.
