# Supplementary material for: One-Shot, One Opportunity: Retrospective Observational Study on Long-Acting Antibiotics for SSTIs in the Emergency Room—A Real-Life Experience
Source: Pathogens. 2025 Aug 6;14(8):781. doi: 10.3390/pathogens14080781 (PMC12389273; doi:10.3390/pathogens14080781)
Supplement: Supplementary file 1 [file pathogens-14-00781-s001.zip › pathogens-3803926-S2.pdf]

Previous antibiotic treatment (n= 11)

Ceftriaxone for 2 days (n= 1),
ceftriaxone + levofloxacin for 2 days (n= 1),
ceftriaxone + daptomycin for 2 days (n= 2),
ceftriaxone + doxycycline for 2 days (n= 1),
amoxicillin/clavulanate for 1 day (n= 1),
amoxicillin/clavulanate for 2 days (n= 3),
amoxicillin/clavulanate for 3 days (n= 1),
amoxicillin/clavulanate + doxycycline for 4 days (n= 1)

Antibiotic in association with LAL (n= 5)

Ceftriaxone (n=1)
Cefixime (n= 1)
Doxycycline (n= 1)
Trimethoprim/sulfamethoxazole (n=1)
Piperacillin/Tazobactam (n= 1)
